# Pro- and Anti-fibrogenic Functions of Gram-Negative Bacterial Lipopolysaccharide in the Liver

**DOI:** 10.3389/fmed.2020.00130

**Published:** 2020-04-21

**Authors:** Chandrashekhar R. Gandhi

**Affiliations:** ^1^Divisions of Gastroenterology, Hepatology and Nutrition, Department of Pediatrics, Cincinnati Children's Hospital Medical Center, Cincinnati, OH, United States; ^2^Cincinnati VA Medical Center, Cincinnati, OH, United States

**Keywords:** stellate cells, activation, fibrosis, endotoxin, LPS, reversal, inflammation

## Abstract

Extensive research performed over several decades has identified cells participating in the initiation and progression of fibrosis, and the numerous underlying inter- and intra-cellular signaling pathways. However, liver fibrosis continues to be a major clinical challenge as the precise targets of treatment are still elusive. Activation of physiologically quiescent perisinusoidal hepatic stellate cells (HSCs) to a myofibroblastic proliferating, contractile and fibrogenic phenotype is a critical event in the pathogenesis of chronic liver disease. Thus, elucidation of the mechanisms of the reversal to quiescence or inhibition of activated HSCs, and/or their elimination via apoptosis has been the focus of intense investigation. Lipopolysaccharide (LPS), a gut-resident Gram-negative bacterial endotoxin, is a powerful pro-inflammatory molecule implicated in hepatic injury, inflammation and fibrosis. In both acute and chronic liver injury, portal venous levels of LPS are elevated due to increased intestinal permeability. LPS, via CD14 and Toll-like receptor 4 (TLR4) and its adapter molecules, stimulates macrophages, neutrophils and several other cell types to produce inflammatory mediators as well as factors that can activate HSCs and stimulate their fibrogenic activity. LPS also stimulates synthesis of pro- and anti-inflammatory cytokines/chemokines, growth mediators and molecules of immune regulation by HSCs. However, LPS was found to arrest proliferation of activated HSCs and to convert them into non-fibrogenic phenotype. Interestingly, LPS can elicit responses in HSCs independent of CD14 and TLR4. Identifying and/or developing non-inflammatory but anti-fibrogenic mimetics of LPS could be relevant for treating liver fibrosis.

## Introduction

Liver fibrosis is a repair response to injury caused by various noxious stimuli such as viral infection (hepatitis B [HBV] and C [HCV] virus), toxins and drugs, autoimmune hepatitis, biliary damage, and copper or iron accumulation. Although fibrosis is reversible, persistent presence of the injury stimulus leads to excessive accumulation of extracellular matrix (ECM), collagens I and III and fibronectin being the major components. This disrupts the hepatic architecture and blood supply to hepatocytes, the site of numerous essential metabolic functions. Ensuing irreversible liver cirrhosis is one of the principal leading causes of morbidity and mortality in the world with organ transplantation as the only option for survival (1–3). Major success has been achieved in treating HCV-induced fibrosis, the most common form of chronic liver disease, through clearance of the virus (4, 5). However, there is alarmingly increasing incidence of alcohol-induced and non-alcoholic (fatty) liver diseases that can remain undiagnosed and thus silently progress to fibrosis/cirrhosis in predisposed individuals (2). Distinct from these are chronic liver diseases originating from the portal tracts (primary biliary cholangitis and primary sclerosing cholangitis) with high morbidity and mortality. Remarkable advancements have been made in identifying the cell types that co-ordinate fibrogenesis as well as the underlying inter- and intra-cellular signaling mechanisms (6–9). Several animal models of liver fibrosis of various etiologies have been developed (10, 11), and mono- and co-culture systems established (8, 12, 13) to discover the mechanisms of cross-communication amongst the liver resident cells, infiltrating inflammatory cells and immune cells implicated in fibrosis at the organ and cellular/subcellular levels. However, fibrosis of the liver and other organs remains untreatable.

It is generally accepted that activated proliferating hepatic stellate cells (HSCs) are responsible for liver fibrosis regardless of the etiology. An exception to this is biliary injury-induced disease in which portal (myo)fibroblasts are the major cells during initial period and are also significantly involved, along with HSCs, at later times of the disease progression (8, 13, 14). Inflammation initiated by the hepatocyte damage plays a critical role both in activation and fibrogenic activity of HSCs. Gut-derived microbial products including Gram-negative bacterial lipopolysaccharide (LPS) enhance inflammation and thus fibrosis during chronic liver injury. Therefore, HSCs have been a topic of intense investigation to discover mechanisms of their responses to inflammatory mediators as well as microbial products. Several lines of enquiry have positively implicated LPS in experimental and human chronic liver disease including non-alcoholic fatty liver disease (NAFLD) (15–18). In contrast, others found inflammatory cytokines, and not serum LPS, to correlate with NAFLD severity (19). However, evidence has emerged showing LPS-induced inhibition of proliferation, reversal of the activated phenotype and mitigation of the fibrogenic activity of HSCs (20–24). This article evaluates such contrasting interactions between LPS and HSCs and discusses the potential of non-inflammatory mimetic(s) of LPS as a therapy for liver fibrosis.

## Disruption of Hepatic Structure and Function In Chronic Liver Injury

The liver receives nearly 70–75% blood from the portal vein and 25–30% from the hepatic artery at the portal triads. The portal veins and hepatic arteries branch after entry into the liver, and eventually the venous and arterial blood mixes up in the capillaries known as “sinusoids.” The sinusoids are lined by specialized endothelial cells with no underlying true continuous basement membrane. The sinusoidal endothelial cells (SECs) possess sieve plates that have pores (~100 nm diameter) called “fenestrations.” The liver-resident macrophages, Kupffer cells, are found within the sinusoids adhering to the endothelial cells, whereas HSCs reside in the “Disse's space” between the SECs and the parenchymal cells (hepatocytes), the main cells responsible for the liver's metabolic function (25). Highly coordinated interactions between the major cell types of the liver (hepatocytes, stellate cells, SECs, Kupffer cells and biliary epithelial cells) via physical contacts and soluble mediators are critical to the liver's physiological functions and maintenance of homeostasis. This balance is disrupted during injury, and persistent injury leads to chronic fibrotic liver disease and its systemic complications.

## Lipopolysaccharide and Liver Injury

**Figure 1:**
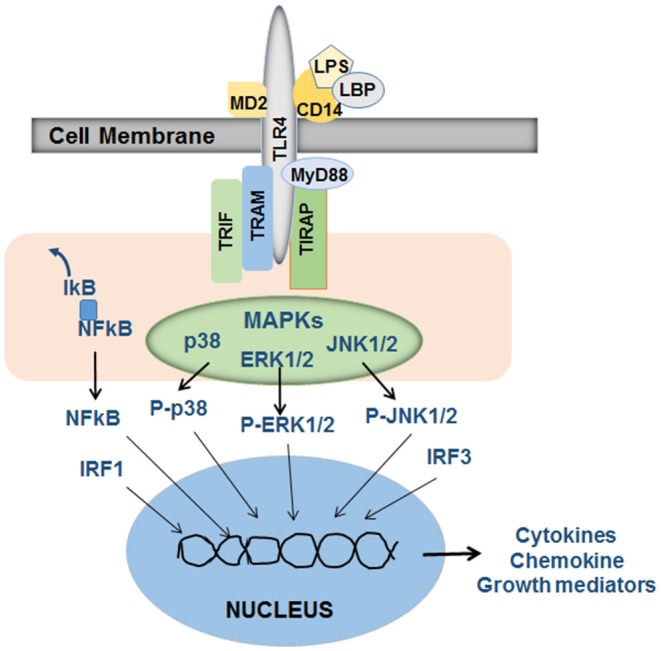
Schematic of LPS-induced signaling in inflammatory cells. LPS in association with LBP (LPS-binding protein) binds to CD14, which then stimulates TLR4 signaling. The adapter proteins MD2 (on the outer side of the cell membrane), and TRIF (TIR-domain-containing adapter-inducing interferon-β), TRAM (TRIF-related adaptor molecule), MyD88 and TIRAP (Toll-Interleukin 1 receptor domain containing adaptor protein) that are associated with the intracellular part of TLR4 are required for LPS-induced and TLR4-stimulated activation of intracellular signaling via NFkB, mitogen activated protein kinases (MAPKs) p38, ERK1/2, and JNK1/2 and well as interferon-regulatory factor (IRF) 1 and 3. Translocation of these activated transcription factors to the nucleus and their subsequent binding to appropriate promoter regions on the DNA instigate transcription of a several cytokines, chemokines and growth mediators specific to a given cell type. The released mediators then act on target cells to promote pathophysiological processes. Adapted from Schwabe et al. (27), Akira et al. (28).

Lipopolysaccharide (LPS), a highly inflammatory endotoxin, belongs to the family of gut-derived microbial products known as pathogen-associated molecular patterns (PAMPs). It is a component of the Gram-negative bacterial cell membrane and is composed of three units, O antigen or O polysaccharide, core oligosaccharide and the active constituent lipid A. LPS mediates its cellular effects through toll-like receptor 4 (TLR4), a type I transmembrane protein with an extracellular leucine-rich repeat domain and a cytoplasmic domain homologous to the cytoplasmic domain of the human interleukin (IL)-1 receptor (26). However, association of lipid A with LPS-binding protein (LBP), a soluble protein that increases the affinity and potency of LPS, is required for the subsequent binding to soluble or membrane-bound CD14. CD14 does not have intracellular domain and therefore the LBP-CD14/LPS complex must bind to TLR4. LPS-induced transmembrane signaling also requires TLR4-associated extracellular (MD2) and intracellular (MyD88, TRAM, TRIF, and TIRAP) adapter components (Figure 1). LPS instigates several signaling cascades (NFkB, interferon-regulatory factors [IRFs], p38, ERK1/2, and JNK mitogen-activated protein kinases, AP1, etc.) in the immune and inflammatory cells coupled to the expression of cytokines including TNF, IL1α, IL1β, IL6, IL10, and type 1 and type 2 interferons (IFNs), chemokines and several other biologically active mediators (27–31) that are critical to liver injury, repair and fibrogenesis. Interestingly, LPS can also elicit cellular response in a MyD88-independent manner (32, 33), and macrophages from Cd14-null mice were shown to produce TNF in response to lipid A (33). Thus, it is important to identify the precise mechanisms of a specific response of a given cell to LPS for better understanding of the pathophysiological processes.

## Multifunctional Hepatic Stellate Cells (HSCs)

**Figure 2:**
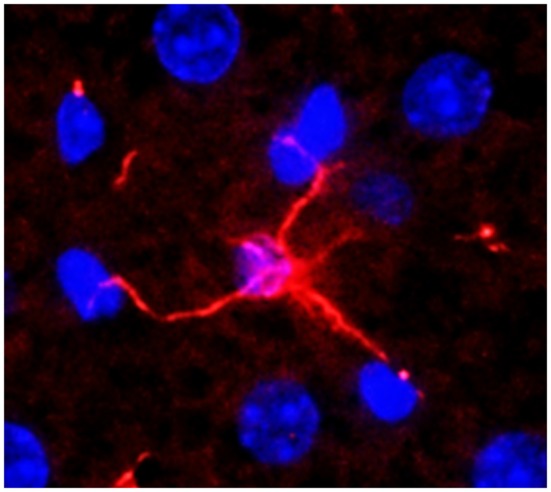
A normal mouse liver section stained for desmin (expressed by hepatic stellate cell, HSC). An HSC can be seen connecting with 4 hepatocytes. Nuclei were stained with DAPI. Adapted from Gandhi (38, 39).

HSCs are located in the Disse's space and are the major storage site of vitamin A within their cytoplasmic lipid droplets (34). HSCs can be identified by vitamin A autofluorescence and expression of cytoskeletal intermediate filament desmin and/or glial fibrillary acidic protein (GFAP). However, zonal expression of these markers as well as retinoid-storage by HSCs is variable (35, 36). It is estimated that about 25% of HSCs may not contain vitamin A (34, 37). Although their cell body is small (~10 μm), HSCs demonstrate physical contact with 2–3 adjacent hepatocytes, SECs and even Kupffer cells and other cells in the sinusoidal lumen via long cytoplasmic processes (38, 39) (Figure 2). Thus, from their strategic location, HSCs are able to influence the functions of almost all hepatic cell types by juxtacrine (contact) and autocrine/paracrine (via released soluble mediators) mechanisms (38–40).

HSCs produce and react with the potent vasoconstrictor endothelin-1 and vasodilator nitric oxide (41–43), which regulate sinusoidal blood flow by inducing HSC contraction and relaxation, respectively (44–46). LPS stimulates the synthesis of both endothelin-1 and nitric oxide by HSCs, and LPS-induced vascular resistance of the previously normal liver is mitigated by endothelin receptor antagonism (47). HSCs also demonstrate remarkable ability to recruit inflammatory and immune cells, and influence their survival and functions (22, 23, 38–40, 48–54). Furthermore, evidence has emerged showing an important role of HSCs in liver regeneration and hepatocellular carcinoma (55–58). With these powerful characteristics, the strategically located HSCs regulate hepatic vascular tone, liver injury and regeneration, and hepatic immunological tolerance.

## Activation of Hscs and Liver Fibrosis

**Figure 3:**
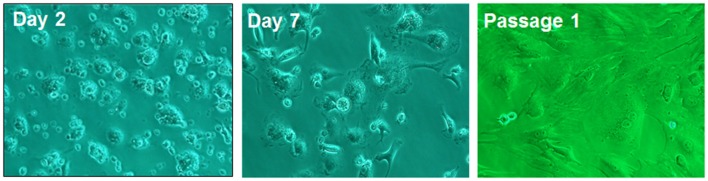
Rat HSCs on day 2, day 7 of culture and in passage 1. On day 2, the cells show typical morphology of quiescent phenotype, and on day 7 of myofibroblast-like phenotype. The passagesd cells are fully activated showing myofibroblastic phenotype. Adapted from Gandhi (38, 39).

Physiologically quiescent HSCs transdifferentiate into highly proliferative, fibrogenic and contractile myofibroblastic activated phenotype (aHSCs) during liver injury (Figure 3). Once activated, HSCs produce excessive amounts of extracellular matrix (ECM) components, which include fibrillary collagens and fibronectin (59). The net deposition of the ECM is regulated by matrix metalloproteinases (MMPs) and tissue inhibitors of metalloproteinases (TIMPs). Kupffer cells are a major source of several MMPs whereas HSCs are the major source of TIMPs. HSCs also produce MMPs. During fibrosis development, the predominance of increased expression of TIMPs and down-regulation of the expression of MMPs (collagenases) is a major cause of progressive ECM deposition. This topic is extensively reviewed by Campana and Iredale (60).

The “initiation” phase of HSC activation begins with the loss of retinoid stores and down-regulation of peroxisome proliferator-activated receptor γ (PPARγ), accompanied by the expression of smooth muscle alpha-actin (αSMA) (via up-regulation of its transcription factor c-Myb) and platelet-derived growth factor β receptor (PDGFβR) (8, 9, 59). It is not entirely clear whether the loss of retinoids is a cause or a consequence of HSC activation. For example, supplementation of the culture medium with retinoic acid retards the rate of HSC activation (61), and the expression of lecithin-retinol acyltransferase (LRAT), an enzyme responsible for the accumulation of retinol as its esters, is lost during HSC activation (62). It was shown that the energy required for activation of HSCs is derived from triglycerides stored in the lipid droplets through autophagic/lysosomal degradation (63). However, mice that lack LRAT are devoid of lipid droplets (62, 64), and yet are similarly susceptible to bile duct ligation (BDL)- or CCl4-induced fibrosis as the wild type (WT) mice, and LRAT-deficient HSCs are similarly activated as WT HSCs (62). Since HSCs are the exclusive cell type to express LRAT in the liver, these results suggest that retinoids may not be absolutely essential for HSC quiescence. Further work is necessary to understand the role of retinoids and triglycerides stored in the lipid droplets in HSC activation.

It is generally accepted that in almost all etiologies of fibrosis, factors derived from injured/dying hepatocytes including apoptotic bodies, danger-associated molecular patterns (DAMPs), reactive oxygen species (ROS) and hedgehog ligands are the initial stimuli for HSC activation (8, 65, 66). High mobility group box 1 (HMGB1), a prominent DAMP released by dying/damaged hepatocytes, is shown to induce activation of HSCs, and also to elicit profibrogenic signals in combination with transforming growth factor-beta 1 (TGFβ) (67). Upon phagocytosis of hepatocyte apoptotic bodies and stimulation with DAMPs, Kupffer cells synthesize and release multiple cytokines, ROS and growth factors such as platelet-derived growth factor (PDGF) that promote activation and proliferation of HSCs (8, 68).

The “initiation phase” is followed by the “perpetuation” phase, as the injury stimulus persists. In this, activated Kupffer cells, modified capillarized SECs, and infiltrating neutrophils and lymphocytes cause HSCs to remain activated and/or cause their further activation and proliferation (8, 9). In this phase, TNFα produced by inflammatory macrophages, including Kupffer cells, stimulates survival signals in HSCs, whereas TGFβ1 induces activation as well as fibrogenic signals. Other cytokines prominently involved in HSC activation, proliferation and fibrosis are IL17, IL1α, and IL1β (67). Importantly, aHSCs themselves produce ROS, pro-inflammatory cytokines and chemokines, and express cell adhesion molecules to recruit circulating inflammatory and immune cells, and retain activated phenotype (8, 9, 69–71). Furthermore, highly activated passaged HSCs and human activated HSC cell line (LX1cells) were shown to increase their expression of αSMA, TGFβ1, and collagen 1a1 upon phagocytosis of hepatocyte-derived apoptotic bodies (65).

Elimination of the injury stimulus causes aHSCs to undergo apoptosis (72), senescence (73), or reversal to quiescent or the so-called “inhibited phenotype” (iHSC) leading to regression of fibrosis (8, 13, 74–76). IL10 and IL22 can be critically involved in the fibrosis reversal process as evidenced by IL10-induced inhibition of the expression of the activation markers in aHSCs (77–79), and IL10- and IL22-induced aHSC death by senescence (80, 81). It is important to note that iHSCs can be rapidly re-activated upon return of the injury stimulus causing accelerated development of fibrosis (75).

In the injured liver Kupffer cells as well as aHSCs are the major source of TGFβ, which is considered to be the most potent cytokine to stimulate ECM synthesis in aHSCs. The autocrine and paracrine stimulation of aHSCs by TGFβ activates the transcription factor complex P-SMAD2/3-SMAD4 (SMAD, small mother against decapentaplegic) and reduced nicotinamide adenine dinucleotide phosphate (NADPH) oxidase-mediated activation of p35-CCAAT/enhancer-binding protein beta (p35-C/EBPβ) (9, 81, 82). Other mediators such as angiotensin II, leptin, ethanol (alcohol) metabolite acetaldehyde and ROS are also major contributors of the synthesis and deposition of excessive amounts of ECM components from aHSCs.

**Figure 4:**
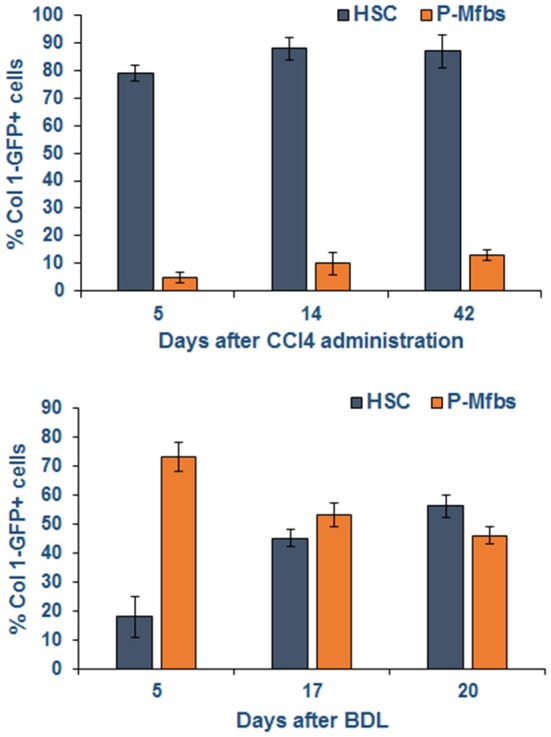
Activated HSCs and P-Mfbs during carbon tetrachloride- or bile duct-ligation-induced liver injury in mice. The cells were identified by flow cytometry using green fluorescence protein (GFP) under collagen 1 promoter. GFP+ and vitamin A+ (HSCs) or GFP+ and vitamin A- cells were separated by flow. Note that HSCs but not P-Mfbs increase in CCl4-induced inflammatory injury and fibrosis, but the number of both cell types increase after BDL. Adapted from Iwaisako et al. (82).

Fas/FasL interactions are also critical to liver injury and fibrosis with an important role of Kupffer cells, which increase the expression of FasL upon phagocytosis of apoptotic bodies (68). Resistance of mice lacking Fas (lpr mice) to injury and fibrosis after bile duct ligation (BDL) (83) indicates that injury to hepatocytes and/or biliary epithelial cells is a critical stimulus for fibrogenesis. As liver injury and fibrosis progress, Fas/FasL interaction can also be a mechanism of limiting fibrosis through apoptosis of aHSCs (84). In contrast, portal myofibroblasts (P-Mfbs) are resistant to Fas/FasL-induced apoptosis (85), indicating their apparent predominance as the fibrogenic cell in biliary injury. It is shown that 5 and 20 days, respectively, after BDL, ~73 and 43% of the fibrogenic cells were found to be activated P-Mfbs as compared to ~18 and 51% aHSCs (82) (Figure 4). In this study, aHSCs and P-Mfbs were distinguished based on the presence (HSC) or absence (P-Mfb) of vitamin A (82), and a significant population of HSCs is devoid of or strongly deficient in vitamin A (34, 35, 37). A comprehensive comparative examination of the mechanisms underlying biliary and other types of liver fibrosis and precise identification of the responsible cells at various stages of its progression will be needed.

## CD14/TLR4-Independent LPS-Induced Inflammatory Response by HSCs

Effective clearance of bacterial endotoxins is an important function of the liver, primarily performed by Kupffer cells and hepatocytes (86–88). Plasma concentrations of LPS and other microbial products are very low or undetectable in physiology, but increase during both acute and chronic liver damage because of increased gut permeability and reduced hepatic clearance (15, 89–92). Gut-derived microbial products are critically involved in complications of endotoxemia occurring in acute and chronic liver injury, HCV infection, obstructive jaundice, cholestasis and chronic alcoholic and non-alcoholic hepatitis (93–98). A recent analysis of the association between serum LPS and chronic liver disease in >6,500 subjects found that serum LPS can be predictive of advanced liver disease (17). Because inflammation is critical to activation and fibrogenic activity of HSCs, LPS has gained much attention as a driver of liver injury, inflammation and fibrosis.

The pro-inflammatory LBP/CD14/TLR4-mediated effects of LPS on Kupffer cells, neutrophils and immune cells implicated in liver damage have been well-characterized (99–101). Activation-dependent response of rat HSCs to LPS by releasing MCP-1 (102) provided evidence for their possible role in hepatic inflammation. In these experiments, high (100 ng/ml) concentration of LPS and serum-supplemented medium were used (102). LPS was later found to stimulate the synthesis of nitric oxide, endothelin-1, TNFα and IL6 in both quiescent and activated rat HSCs at concentration as low as 1–10 ng/ml in serum-free condition (20, 21, 43, 103). This indicated that rat HSCs respond to LPS independent of CD14/TLR4 as serum is the source of LBP, which is produced by hepatocytes but not HSCs (21). These findings are of significant importance because LPS causes liver injury in CD14-independent manner (104); LPS-induced production of TNFα and IL6 in wild type (WT), TLR4-knockout (KO) and CD14-KO mice was similar following partial hepatectomy (105); and bile duct ligation or CCl4 administration elicited similar liver injury in WT, TLR4-mutant (C3H/HeJ) or TLR4-KO mice (23, 106). LPS also elicited similar inflammatory response in HSCs from WT and TLR4-KO (23) or CD14-KO mice (107). Interestingly, although quiescent rat and human HSCs possess very low (negligible) expression of TLR4, LPS induced NFkB activation and stimulated the synthesis of inflammatory cytokines in rat (21) but not human (108) quiescent HSCs (qHSCs). Whereas, both rat and human HSCs express TLR4 upon activation (21, 108), mouse qHSCs contain abundant expression of TLR4 (106). These findings indicate species-specific differences in CD14/TLR4-dependence or -independence of LPS effects may have important implications in hepatic pathophysiology.

In addition to the pro-inflammatory cytokines and chemokines, LPS also stimulates secretion of anti-inflammatory cytokines such as IL10 from HSCs (51). Transcriptomic analysis demonstrated that the repertoire of factors expressed by rat aHSCs and modulated by LPS was much extensive and included numerous cytokines/chemokines, cell adhesion molecules, signal transduction factors, as well as growth mediators (22). Obviously, the direct actions of LPS on HSCs are of critical importance in acute and chronic liver injury.

## PRO- and Anti-Fibrogenic Effects Of LPS On HSCs

As described above, inflammation, initiated by apoptotic bodies, DAMPs and cytokines released by injured/dying hepatocytes, plays a critical role in HSC activation and liver fibrosis. With continued presence of the injury stimulus, dying hepatocytes, Kupffer cells, recruited lymphocytes and even HSCs contribute to the persistent inflammatory environment. The role of Kupffer cells in hepatic inflammation and fibrosis has been investigated extensively, and depletion or blockade of Kupffer cells with gadolinium chloride was found to mitigate liver fibrosis in several murine models of liver injury including that by CCl4, dimethylnitrosamine and BDL (109–111). There is also evidence for a crucial role of the recruited blood-derived macrophages in liver fibrosis and their switch to anti-inflammatory (restorative) phenotype during its resolution (112, 113). These restorative macrophages may induce apoptosis of aHSCs or their reversal to either quiescent or inhibited phenotype (75, 114).

**Figure 5:**
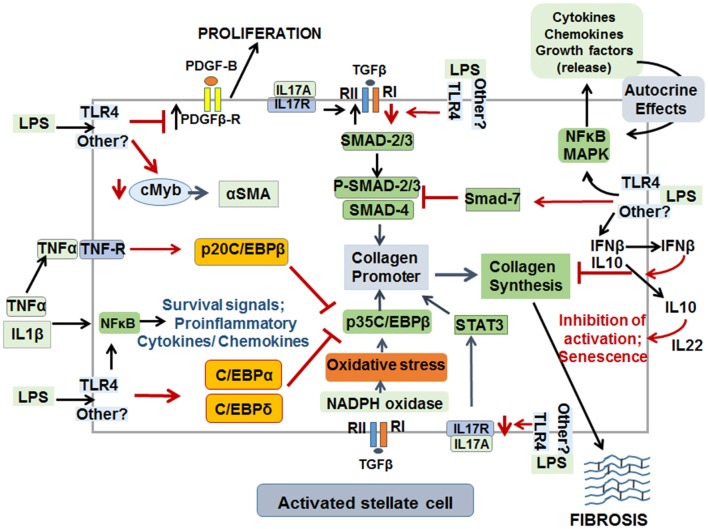
Schematic representation of pro- and anti-fibrogenic effects of LPS on activated HSCs. Although LPS may act through TLR4 on aHSCs, it can also exert effects in a non-TLR4 pathway that has not been identified yet. LPS by stimulating synthesis of several cytokines and chemokines, via stimulation of NFkB and MAPK pathways, promotes survival (TNFα and IL1β) in an autocrine manner. The mediators released thus can also stimulate ECM synthesis, migration and proliferation of aHSCs. LPS down-regulates cMyb transcription factor and thus reduces the expression of α-SMA, a major marker of aHSCs. By down-regulating PDGFβR, LPS mitigates proliferation of aHSCs. LPS inhibits TGFβ-induced ECM synthesis by down-regulating TGFβR1, by increasing expression of SMAD7, C/EBPα, C/EBPδ, and p20C/EBPβ. IL17 can act directly on HSCs to stimulate ECM synthesis and by up-regulating TGFβRII. However, by down-regulating the expression of IL17R, LPS can reduce IL17-induced ECM synthesis by aHSCs. Finally, increased production of IL10 and IFNβ by LPS-stimulated aHSCs can be a mechanism of inhibition of ECM synthesis, activation and promotion of senescence.

Although LPS has been implicated in liver fibrosis through its pro-inflammatory effects, whether its direct actions on HSCs has a role in fibrogenesis has remained relatively unexplored. LPS was found to inhibit DNA synthesis, concentration-dependently, in activated rat HSCs in presence or absence of serum, the source of LBP (20, 21). This observation is intriguing as activation and proliferation of HSCs are essential components of the initiation and progression of fibrosis. Recent work by Sharma and coworkers confirmed that LPS inhibits proliferation of culture-activated aHSCs as determined by Ki67 labeling *in vitro*, and even HSCs isolated from LPS-treated CCl4-induced chronically injured liver showed size reduction and reduced Ki67 labeling as compared to the cells from rats that did not receive LPS (24). This effect of LPS *in vivo* is impressive since hepatic inflammation was augmented, and indicated that LPS may arrest or mitigate HSC proliferation to limit ongoing fibrosis development in the inflammatory environment (i.e., in the presence of injury stimulus). On the other hand, LPS stimulates NFkB activation (a pro-inflammatory and pro-survival pathway) in HSCs (23, 103, 108), importance of which was confirmed by the observation showing reduced hepatic fibrogenesis after NFkB inhibition (114). It is apparent that such contemporaneous stimulation of the opposing signaling pathways can be of significant importance in regulating expansion of HSCs in the fibrotic liver (see Figure 5 for schematic of opposing effects of LPS on HSC activation and fibrosis).

LPS-induced inhibition of DNA synthesis in qHSCs (21) suggested that it may not have direct effect on their activation. However, LPS-preconditioned qHSCs are activated upon incubation with TGFβ or when co-cultured with Kupffer cells (106), a main source of TGFβ in the liver (110, 115, 116). LPS was found to down-modulate the expression of BMP and activin membrane-bound inhibitor (BAMBI), a pseudoreceptor for TGFβ1, in qHSCs thereby sensitizing them to TGFβ1-induced activation and fibrogenic activity (106). In this regard, Kupffer cells were shown to become more sensitive to the effects of LPS after bile duct ligation and exhibited significant increase in phagocytic activity, oxidative burst, and cytokine production (117). Kupffer cells isolated from LPS-injected mice were reported to show increased expression of TNFα, IL6 and TGFβ (118), and at high concentrations, LPS promotes autophagy/lipophagy, down-regulates BAMBI and enhances TGFβ1 signaling in activated HSCs and HSC cell line (119). However, LPS does not stimulate the synthesis of TGFβ in purified HSCs (22, 23), and LPS administration to naïve mice also does not increase the expression of TGFβ (23). Furthermore, hepatic expression of BAMBI was not altered in mice that received chronic CCl4 treatment or in LPS-challenged culture-activated HSCs, but it was down-regulated in the livers of naïve mice upon acute LPS treatment (23). Because HSCs (and not hepatocytes or Kupffer cells) express BAMBI (106), these data suggest that its down-regulation occurring early during liver injury may not be sustained in the chronic phase. On the contrary, up-regulation of TGFβ-receptors in aHSCs (23, 24) could be a more dominant mechanism of liver fibrosis. TGFβ1 may also self-regulate its effects by modulating BAMBI expression. For example, TGFβ1 causes up-regulation of BAMBI mRNA and protein in HEPG2 cells via the P-SMAD2/3-4 transcriptional pathway (119), and stimulation of WNT/β-catenin signaling increases BAMBI in colorectal tumor cells (120). Since LPS increases nuclear accumulation of β-catenin in human hepatoma cell lines (121), it will be important to determine whether LPS ± TGFβ1 induce SMAD and/or Wnt/β-catenin signaling in qHSCs or aHSCs and regulate BAMBI, TGFβ-R1 and TGFβ-R2 expression both *in vivo* and *in vitro* for better understanding of the pathway: LPS → Kupffer cells/HSCs → BAMBI → TGFβ1 → activation of HSCs/fibrosis.

At 21 days after BDL, hepatic fibrosis was reported to be 30-50% less in CD14-deficient and in LBP-deficient mice than in the WT mice (122). However, there was no difference in lymphocyte and neutrophil infiltration but activation of macrophages was lower in CD14-KO mice as determined by the expression of Cd11b, a component of the C3 complement receptor primarily expressed on myeloid cells (i.e., macrophages and monocytes) (123). Saito and coworkers proposed that depletion of neutrophils does not have significant effect on BDL-induced fibrosis but LPS-stimulated Kupffer cells enhance hepatic fibrogenesis (123). The TLR4 mutant (C3H/HeJ) mice were also found to show much less CCl4- or BDL-induced fibrosis as compared to the WT mice (106). In contrast, C57BL/6J (B6-WT) and B6.B10ScN-Tlr4^*lps*−*del*^/JthJ (TLR4-KO) mice demonstrated similar susceptibility to CCl4-induced fibrosis as analyzed by Sirius red staining, collagen I expression and hydroxyproline concentration, although necroinflammation and liver injury were lower in the latter (23). The expression of TNFα and CXCL1 increased similarly in CCl4-treated WT mice and TLR4-KO mice but that of antifibrogenic IFNγ increased only in WT mice (23). Furthermore, the expression of αSMA and the number of desmin-positive cells increased similarly in CCl4-treated WT and TLR4-KO mice suggesting that TLR4 activation is not necessary for activation and proliferation of HSCs. It is apparent that hepatocyte injury-induced activation of Kupffer cells and HSCs and also inflammation are more relevant to HSC activation and fibrosis. While these data demonstrate that LPS/TLR4 interaction may not be critical to fibrosis development in chronic liver disease, activation of TLR4 as well as TLR5, TLR7, and TLR9 was actually found to be beneficial in chronic hepatitis B virus infection by reducing the viral replication (124). Such effects of LPS and other PAMPs on TLRs can be self-limiting mechanisms of chronic liver disease in majority of HBV-infected subjects.

In contrast to the down-modulatory effect of LPS on aHSCs (*in vivo* and *in vitro*), augmentation of CCl4-induced liver fibrosis in mice was reported within a very short time of just 4 h following administration of 10 mg/kg LPS (125). LPS administration (0.5 mg/kg; 3 times a week) from the beginning of NASH-inducing choline-deficient L-amino acid-defined (CDAA) diet in mice was also reported to increase inflammation, activation of HSCs and pericellular fibrosis (126). It should be noted that CDAA diet does not cause obesity or insulin resistance in rats, in contrast to mice that develop obesity and insulin resistance and limited fibrosis (127). Nevertheless, LPS effect described above contradict our observations that CCl4-induced hepatic fibrosis is not altered at 24 h after intraperitoneal administration of 5 mg/kg LPS (24). However, αSMA expression was strongly reduced by LPS *in vivo*, and HSCs isolated from LPS-treated CCl4-fibrotic rats showed reduced size, proliferation and expression of Acta 2, cMyb, PDGFβR, TGFβR1, Col1a1, and fibronectin but increased expression of TNFα, IL6, CXCL1 (24). CCl4-induced liver fibrosis was also not affected by a weakly inflammatory lipid A-derivative monophosphoryl lipid A although it caused reduction in αSMA expression in HSCs both *in vivo* and *in vitro* (24). In regard to whether LPS is really critical in promoting or mitigating fibrogenesis, antibiotic treatment of mice was found to reduce BDL- as well as CCl4-induecd fibrosis (106). However, much stronger CCl4-induced fibrosis was observed in germ-free mice as well as Myd88/Trif-deficient mice compared to the WT mice (128). Furthermore, repopulation of Gram-negative microbes (*E. coli*, the source of LPS) following dysbiosis did not affect fibrosis when compared to mice that did not receive *E. coli* (128). A recent investigation also reported that monocytes-derived macrophages stimulated with LPS and monosodium urate increase MMP3 and MMP9 in aHSCs and down-modulate pro-fibrogenic markers (129). These data and the observations showing unique interactions between HSCs and LPS (20–23) suggest that LPS has a dual role as a promoter of liver fibrosis by causing inflammation, and contemporaneously limit fibrosis by its direct effects on aHSCs.

## Other LPS-Stimulated Pathways Regulating Liver Fibrosis

The livers of chronically CCl4-treated rats were found to contain several apoptotic aHSCs, which increased further when oxidative stress was induced by administration of tert-butylhydroperoxide (130, 131). *In vitro* experiments confirmed that oxygen-free radicals cause apoptosis of aHSCs (130). Thus, although LPS-stimulated synthesis of free radicals in Kupffer cells (99) and HSCs (103) are generally considered as pro-fibrogenic, the same molecules appear to instigate signaling mechanism of cell death and prevent aHSC proliferation and fibrogenic activity. Along the same line, although autocrine or paracrine actions of TNFα on aHSCs provide NFkB activation-induced cell survival mechanisms, TNFα also stimulates binding of p20C/EBPβ and C/EBPδ to Cola1 promoter and thus represses p35C/EBPβ-induced transcription and fibrosis (132, 133). Interestingly, TGFβ1 has been shown to induce and increase SMAD7 (an inhibitor of pro-fibrogenic P-SMAD2/3) in several cell types including HSCs (134–136). This suggests a feed-back inhibition of pro-fibrogenic action of TGFβ1 in aHSCs. LPS increases SMAD7, and p20C/EBPβ and C/EBPδ (inhibitors of p35C/EBPβ) expression and down-regulates cMyb (a transcription factor for αSMA) expression in aHSCs *in vivo* and *in vitro* (24). Because LPS also strongly stimulates TNFα synthesis by HSCs (20–23, 103), the autocrine loop of its action on inhibitory C/EBP pathway might be a limiting mechanism of fibrogenesis (Figure 5).

IL17A promotes not only activation of inflammatory cells, but also stimulates collagen synthesis by HSCs through activation of signal transducer and activator of transcription 3 (STAT3) (137). In contrast, another study reported that IL17 does not directly cause activation of HSCs or induce fibrogenic response, but increases TGFβRII expression in HSCs sensitizing them to TGFβ1/SMAD2/3-induced collagen 1 synthesis (138). LPS down-regulates TGFβR in aHSCs (22, 23), and does not affect IL17A expression although it increases gene transcript of IL17F by more than 10-fold (22). Because IL17A and IL17F share the same receptors (IL17Ra and IL17Rc) (139), a similar fibrogenic effect of IL17F via autocrine pathway in HSCs may not be ruled out. However, microarray analysis showed robust decrease in Il17ra and Il17re in aHSCs stimulated with LPS (22). These findings indicate that LPS-induced down-regulation of both TGFβR and IL17R may limit fibrogenesis during chronic liver injury.

While pro-inflammatory and pro-fibrogenic mediators are produced by various cells during chronic liver injury, there is also abundant evidence for contemporaneous generation of anti-inflammatory and anti-fibrogenic factors such as IL10 and IL13. IL10-KO mice show increased neutrophil infiltration and hepatic fibrosis during repeated CCl4 administration (140). Kupffer cells produce IL13 and not IL10 under basal conditions, and LPS stimulates secretion of IL10 but not of IL13 from them (141–143). HSCs also produce IL10 spontaneously, which is strongly stimulated by LPS (22, 50). Such increased production of IL10 and IL13 can be yet another pathway of limiting liver fibrosis.

IL22 is an interesting cytokine that can be a part of anti-fibrotic mechanisms due to its ability to promote senescence and apoptosis of aHSCs both *in vivo* and *in vitro* (80). These effects of IL22 were found to be mediated via the activation of STAT3 and suppressor of cytokine signaling 3 (SOCS3) (144). However, STAT3 activation was also reported to be a mechanism of IL17-induced collagen synthesis by aHSCs via an IL6-dependent autocrine pathway, and deletion of IL22 exacerbated CCl4- as well as BDL-induced fibrosis (137). Furthermore, leptin-induced JAK2/STAT3 activation increased ECM synthesis and thereby fibrosis, and SOCS-3 activation negatively regulated JAK/STAT signaling (144).

Interferons (IFN) are a family of natural glycoproteins with antiviral activity, and type I IFNs (IFNα and IFNβ) have been widely used for viral eradication in patients with chronic viral hepatitis (145, 146). IFNα treatment was found to resolve liver fibrosis by causing significant reduction in the number of aHSCs (147–151). IFNβ was also shown to exhibit antifibrotic property and has been used to treat chronic HCV infection (150, 151). Recombinant human IFNβ decreased the expression of αSMA, collagen I and III, TGFβ1, PDGF-BB and SMAD4 in culture-activated rat or human HSCs, and increased SMAD7 expression (152). LPS stimulates IFNβ expression in HSCs (22, 49, 52) and it is likely that this can be an autocrine-inhibitory loop to reduce fibrosis. Interestingly, HSC-released IFNβ was found to be a major cytokine to cause autophagy in hepatocytes as a cell survival mechanism (Figure 5), but it could also induce acute liver injury through activation of IRF1 signaling in mice upon concanavalin A challenge (49, 50, 52).

## Perspective

The well-orchestrated communications between the various liver cell types maintain the physiological function of the organ despite exposure to numerous toxic substances, microbial and viral products, food- and environ-derived antigens, and drugs and xenobiotics on a regular basis. During liver injury, this mechanism is disrupted with an immediate repair response that involves activation of HSCs and/or P-Mfbs, resulting in increased production of ECM causing liver fibrosis. This mechanism involves mediators produced by the resident cells (hepatocytes, Kupffer cells, endothelial cells and cholangiocytes) as well as recruited inflammatory and immune cells. Upon termination of the injury stimulus, fibrosis is resolved and the system returns back to the physiologic state. However, persistence of injury stimulus causes progression of fibrosis to cirrhosis and, in some cases, hepatocellular carcinoma. The liver also has a remarkable ability to produce mediators that instigate mechanisms of resistance to fibrosis. Although the levels of a highly pro-inflammatory endotoxin (LPS) are elevated, and it has been implicated in fibrosis progression, evidence also indicates that it can reverse the activated fibrogenic phenotype of HSC to non-fibrogenic phenotype. It is of interest that LPS can exert this effect in absence of CD14/TLR4, which is essential for the generation of pro-inflammatory cytokines and chemokines from cells such as Kupffer cells, monocyte, and neutrophils. Development of LPS mimetics that do not engage CD14/TLR4 but still can act on activated HSCs will be a novel way to reverse these cells to the non-fibrogenic phenotype for treating liver fibrosis.

## Synopsis

This article describes the pro-fibrogenic as well as antifibrogenic effects of Gram-negative bacterial endotoxin lipopolysaccharide (LPS). This highly pro-inflammatory mediator is implicated in liver injury, inflammation, and fibrosis of various etiologies. Experiments using animal models of liver fibrosis and isolated cells showed that LPS stimulates synthesis of cytokines including TNFα, IL6, IL1β, and PDGF in Kupffer cells and infiltrating inflammatory and immune cells. These mediators cause activation and proliferation of the fibrogenic hepatic stellate cells (HSCs). In response to mediators such as TGFβ released by Kupffer cells and HSCs themselves, HSCs produce extracellular matrix (ECM) components (collagen I, collagen III, fibronectin) causing fibrosis of the liver. In contrast, LPS acts on activated HSCs directly and reduces the expression of the activation marker α-SMA through down-regulation of its transcription factor for cMyb. LPS also increases expression of SMAD7, p20-C/EBPβ, C/EBPα and C/EBPδ in activated HSCs, which are inhibitors of pro-fibrogenic signaling induced by TGFβ and other pro-fibrogenic mediators. Furthermore, LPS down-regulates TGFβR1 expression in activated HSCs thus mitigating TGFβ-induced fibrogenic activity. LPS stimulates the synthesis of anti-fibrogenic cytokines type 1 interferons and IL10 in HSCs. LPS also stimulates the synthesis of TNFα in HSCs and Kupffer cells. While TNFα is a pro-inflammatory cytokine that promotes survival of HSCs, it also stimulates p20-C/EBPβ and C/EBPδ that block p35C/EBPβ-induced ECM synthesis. The down-modulation of the markers of activation and fibrosis is observed in the HSCs isolated from the fibrotic liver treated *in vivo* with LPS. However, the short-term 24 h treatment with LPS *in vivo* increases inflammation and does not cause reduction in fibrosis. Finally, the antifibrogenic effects of LPS can be mimicked by its weakly inflammatory mimetic monophosphoryl lipid A. Such opposing effects of LPS can be potentially important in limiting liver fibrosis.

## Author Contributions

CG is responsible for the concept and writing of this manuscript.

## Conflict of Interest

The author declares that the research was conducted in the absence of any commercial or financial relationships that could be construed as a potential conflict of interest.

## Abbreviations

AP1: Activator protein 1
BAMBI: BMP and activin membrane-bound inhibitor
CCl4: carbon tetrachloride
C/EBP: CCAAT/enhancer-binding protein
ECM: extracellular matrix
ERK: extracellular-signal-regulated kinase
GFAP: glial fibrillary acidic protein
HBV: hepatitis B virus
HCV: hepatitis C virus
HSC: hepatic stellate cell
IFN: interferon
IL: interleukin
IRAK: Interleukin-1 receptor-associated kinase-like
IRF: interferon-regulatory factor
JNK: c-Jun N-terminal kinases
KO: knockout
LBP: lipopolysaccharide-binding protein
LPS: lipopolysaccharide
MAPK: mitogen-activated protein kinase
MMP: matrix metalloproteinase
NADPH: reduced nicotinamide adenine dinucleotide phosphate
NFkB: nuclear factor kappa-light-chain-enhancer of activated B cells
PAMP: pathogen-associated molecular pattern
PDGF: platelet-derived growth factor
P-Mfb: periportal myofibroblasts
PPAR: peroxisome proliferator-activated receptor
PRR: pattern recognition receptor
ROS: reactive oxygen species
SEC: sinusoidal endothelial cell
SMAD: small mother against decapentaplegic
TGF: transforming growth factor
SOCS: suppressor of cytokine signaling
STAT: Signal transducer and activator of transcription
TIMP: tissue inhibitor of metalloproteinases
TIR: Toll/interleukin-1 receptor
TIRAP: TIR domain containing adaptor protein
TLR: toll-like receptor
TNF: tumor necrosis factor
TRAIL: TNF-related apoptosis-inducing ligand
TRAM: TRIF-related adaptor molecule
TRIF: TIR-domain-containing adapter-inducing interferon-β)
WT: wild type.
